# Impact of maternally derived immunity on piglets’ immune response and protection against porcine circovirus type 2 (PCV2) after vaccination against PCV2 at different age

**DOI:** 10.1186/s12917-016-0700-1

**Published:** 2016-05-11

**Authors:** Paolo Martelli, Roberta Saleri, Giulia Ferrarini, Elena De Angelis, Valeria Cavalli, Michele Benetti, Luca Ferrari, Elena Canelli, Paolo Bonilauri, Elena Arioli, Antonio Caleffi, Heiko Nathues, Paolo Borghetti

**Affiliations:** Department of Veterinary Science, University of Parma, Via del Taglio, 10 - 43126 Parma, Italy; Istituto Zooprofilattico Sperimentale della Lombardia e dell’Emilia Romagna (IZSLER), Via Pitagora, 2-42100 Reggio, Emilia Italy; Veterinary Practitioner, 46100 Mantua, Italy; Swine Clinic, Department of Clinical Veterinary Medicine, Vetsuisse Faculty, University of Bern, Bremgartenstrasse 109a, CH-3012 Bern, Switzerland

**Keywords:** *Porcine circovirus* type 2 (PCV2), Vaccine efficacy, Protection, Immune response

## Abstract

**Background:**

This study was aimed at evaluating the clinical protection, the level of *Porcine circovirus* type 2 (PCV2) viremia and the immune response (antibodies and IFN-γ secreting cells (SC)) in piglets derived from PCV2 vaccinated sows and themselves vaccinated against PCV2 at different age, namely at 4, 6 and 8 weeks. The cohort study has been carried out over three subsequent production cycles (replicates). At the start/enrolment, 46 gilts were considered at first mating, bled and vaccinated. At the first, second and third farrowing, dams were bled and re-vaccinated at the subsequent mating after weaning piglets. Overall 400 piglets at each farrowing (first, second and third) were randomly allocated in three different groups (100 piglets/group) based on the timing of vaccination (4, 6 or 8 weeks of age). A fourth group was kept non-vaccinated (controls). Piglets were vaccinated intramuscularly with one dose (2 mL) of a commercial PCV2a-based subunit vaccine (Porcilis® PCV). Twenty animals per group were bled at weaning and from vaccination to slaughter every 4 weeks for the detection of PCV2 viremia, humoral and cell-mediated immune responses. Clinical signs and individual treatments (morbidity), mortality, and body weight of all piglets were recorded.

**Results:**

All vaccination schemes (4, 6 and 8 weeks of age) were able to induce an antibody response and IFN-γ SC. The highest clinical and virological protection sustained by immune reactivity was observed in pigs vaccinated at 6 weeks of age. Overall, repeated PCV2 vaccination in sows at mating and the subsequent higher levels of maternally derived antibodies did not significantly interfere with the induction of both humoral and cell-mediated immunity in their piglets after vaccination.

**Conclusions:**

The combination of vaccination in sows at mating and in piglets at 6 weeks of age was more effective for controlling PCV2 natural infection, than other vaccination schemas, thus sustaining that some interference of MDA with the induction of an efficient immune response could be considered. In conclusion, optimal vaccination strategy needs to balance the levels of passive immunity, the management practices and timing of infection.

## Background

The control of *Porcine circovirus* type 2 (PCV2) associated diseases (PCVD) is based on management strategies, control of coinfections, and vaccination. At present, different commercial PCV2 vaccines are available, licenced for use in piglets most often from three weeks of age and all of them have been described to be effective in controlling the disease [4, 6, 9, 15, 29]. In fact, the PCV2 vaccines are able to reduce the viral burden and viral-induced specific lesions in the lymphoid tissue, but the potential interference of vaccines with maternally derived immunity and the optimization of the vaccination timing are still worth investigating. Deeper understanding is required with regard to the age-related maturity and responsiveness of piglets’ immune system towards PCV2 vaccines and the extent of passive immunity either from sows’ PCV2 infection or vaccination. Several studies showed that vaccination against PCV2 in piglets with high levels of maternally derived antibodies (MDA) can result in clinical protection [4, 9, 14, 15, 22]. However, in some of these studies, maternal immunity apparently interfered with the humoral immune response after vaccination [9, 22]. Most of the authors characterised the humoral immunity towards PCV2 by determination of total PCV2-specific antibodies and/or by demonstration of an increase of the concentration showing seroconversion that occurs either in subclinically infected or clinically affected pigs, e.g. PMWS-affected pigs [10, 26, 30]. Apart from the humoral immunity, previous reports based on laboratory trials demonstrated that cell-mediated immunity measured as levels of PCV2-specific interferon-γ (IFN-γ) secreting cells (SC) may play a role in mediating viral clearance in combination with neutralising antibodies and that the intensity of the cell-mediated immune response may be influenced by the load and the extent of viral replication [7, 18, 19]. These features were investigated under field conditions both in diseased pigs naturally infected with PCV2 in combination with coinfections and in vaccinated animals showing no or only a few clinical signs [5, 16, 23, 31].

Previous studies showed that high levels of MDA in piglets do not allow eliciting seroconversion after vaccination. Conversely, when the level of passively acquired antibodies is lower than a certain titre (exact value is depending on the serological method), the vaccine administration induces a marked seroconversion [8, 9, 15, 16]. Usually, in piglets vaccinated in the face of a high level of passive immunity, a decline of antibody is observed similarly to the decline in unvaccinated animals. Moreover, Haake and others [11] suggested that vaccination at one week of age clearly affects the induction of an active humoral immunity in piglets. In contrast, the cell-mediated immune response is not affected by MDA levels and contributes to the long-lasting protection [5, 7, 8, 15].

This study was aimed at investigating the clinical protection (vaccine efficacy) and the immune responses (both humoral and cell-mediated) of a one-dose PCV2 subunit vaccine administered in piglets. In particular, identifying the ideal time of vaccination (4, 6 or 8 weeks of age), also in respect to their levels of passively acquired immunity being derived from gilts and sows repeatedly vaccinated at mating against the same antigen, was of interest. The outcome parameters were comprised by clinical (morbidity and mortality), productive (average daily weight gain - ADWG), and virological (PCV2 viremia and viral load) parameters. The immune response in piglets was assessed in terms of development of humoral (total antibodies) and cell-mediated (PCV2-specific IFN-γ secreting cells) immunity.

## Methods

### Clinical history of the herd

The study was carried out in the Northern part of Italy at a farm selected with regard to its history of PCVD. The farm was constituted of a 1200-sow farrow-to-finish herd that, in the previous years, had experienced mortality in the nursery and fattening periods due to PCVD. This diagnosis was based on clinical signs, gross lesions, histopathological findings, and presence of PCV2 in lesions of the lymphoid tissue, all together typical for PCVD in accordance to Sorden [32]. Seropositivity to PCV2 in all categories of animals (replacement gilts, sows, nursery, growing and finishing pigs) was demonstrated by enzyme-linked immune sorbent assay (ELISA). Since the occurrence of the disease, the piglets have been vaccinated at three weeks of age, whereas gilts and sows were vaccinated at each mating. At the time of start of the study no clinical signs of PCV2-SD (PCV2-systemic disease; [28]) were noted. Diagnostic investigations performed on dead pigs older than 15 weeks of age demonstrated no PCV2-specific lesions in the lymphoid tissue. Instead very low titres of the virus determined by PCR were suggestive for PCV2 subclinical infection in several pigs (PCV2-SI).

The involvement of *Porcine reproductive and respiratory syndrome virus* (PRRSV) in the clinical problems of the herd was demonstrated by repeated detection of the virus by PCR in nursery and growing pigs that were suffering from respiratory disease. However, based on diagnostics the herd had been classified as “positive - stable” for PRRS according to Holtkamp and others [13]. The presence of the most common secondary bacteria (*Streptococcus* spp., *Pasteurella multocida*) was confirmed in animals also tested infected by PRRSV. The farm was seronegative for *Aujeszky’s disease virus* (ADV) and *Actinobacillus pleuropneumoniae* and seropositive for *Mycoplasma hyopneumoniae*. Sows were vaccinated against ADV (three times per year), *Porcine parvovirus*, *Erysipelothrix rhusiopathiae* (both at mid-lactation) and *Swine influenza virus* (SIV). Piglets were vaccinated against *M. hyopneumoniae* once at one week of age and ADV according to the National Control Program of Italy.

### Experimental design

This study was a double-blind, randomised, controlled field trial performed in accordance to the principles of *“*Good Clinical Practice*”* with the consent of animals’ owner and the protocol had been previously evaluated and approved by the Ethical Committee for Animal Experimentation of the University of Parma (23/12). The work has been carried out over three subsequent production cycles (replicates). At the beginning, 46 commercial hybrid gilts at 230 to 260 days of age, were included at their first mating. Replacement gilts were produced into the farm. They were individually identified by numbered ear tagging and a blood sample of approx. 10 mL was obtained by puncture of the *Vena jugularis externa* from each animal. Subsequently the gilts were vaccinated with one dose (2 mL) of a commercial PCV2a-based subunit vaccine (Porcilis® PCV, MSD Animal Health, Boxmeer, The Netherlands) via intramuscular injection in the neck (off-label use). At day 113 of the first, second and third gestation period, these animals were bled; i.e. two days prior to expected farrowing date. The sows were re-vaccinated against PCV2 at the same day of subsequent mating after their piglets had been weaned. The day of inclusion of suckling pigs was at their weaning (D -1). On this day, suckling pigs of each farrowing (first, second and third parity) were identified, double ear-tagged (both ears) and randomly assigned to one of the treatment groups. The treatment groups with 100 animals each differed in the time when vaccination was applied to the piglets. Namely, animals in the first group were vaccinated at four weeks of age (weaning age) (group A), six weeks of age (group B) or eight weeks of age (group C). One group of piglets was kept non-vaccinated (group D) as placebo/control. The animals were assigned to the different groups as they came to hand sequentially using a randomized block design (*n* = 4) across litters. The identification of the sow and the date of birth of the piglets were recorded.

The piglets were vaccinated with 2 mL of a commercial PCV2 vaccine (Porcilis PCV®) administered intramuscularly according to the manufacturer’s recommendations. The non-vaccinated control pigs received the same amount of adjuvant (2 mL) by injection in the neck at four weeks of age. The recording of the clinical signs was performed by a veterinarian, who was blinded regarding the treatments. At weaning, all piglets under investigation were moved to the nursery unit and kept commingled in pens of 25 pigs per pen each, all constructed in a unique barn. Upon each accommodation change, pigs were commingled according to usual farm procedures. Treatments, housing, husbandry and feeding were in accordance to the European Union Guidelines on Animal Husbandry and were identical for all experimental groups.

During the first week post-vaccination, pigs were monitored daily for side reactions. At weaning (four weeks of age) and at vaccination (four, six or eight weeks of age), blood samples were collected from 20 piglets per treatment group. These piglets were identified by their number and a differently coloured ear tag. Further sets of blood samples were then taken every four weeks until slaughter (eight [group A only]), 12, 16, 20, 24, 28 and 32 weeks of age in groups A and C, and 10, 14, 18, 22, 26, 30 and 34 in group B). The 20 pigs of the control group were blood sampled four weeks after they had received only the adjuvant (at the time of weaning) and then on a two week interval until slaughter (10, 12, 14, 16, 18, 20, 22, 24, 26, 28, 30, 32 and 34 weeks of age). The same procedures were applied in all three replicates (litters).

### Clinical signs (morbidity) and mortality recording

The individual treatment of every pig was recorded on a daily basis and considered as a parameter for the measurement of morbidity. Dead pigs or those that had to be euthanized for reasons of animal welfare were recorded and immediately submitted to pathological investigations.

### Body weight and average daily weight gain (ADWG)

Individual body weights of the pigs enrolled in the study were measured at inclusion, at 12 and at 24 weeks of age. The ADWG was calculated based on the daily weight gain of all the animals being alive at the end of each weighing period.

### Gross pathology and histopathology

All pigs that died spontaneously and those needing euthanasia underwent gross pathological examination and histopathology within 24 h after death. Samples from inguinal, mesenteric and mediastinic lymph nodes were removed and fixed in 10 % buffered formalin. For routine histopathology, 5-μm thick sections of the lymph nodes were stained with haematoxylin-eosin and examined for lesions compatible with PCV2-SD. The diagnosis of PCV2-SD was concluded, when all the three criteria for the disease were present [28] including moderate-to-high amounts of PCV2 in the lesions determined by quantitative real-time PCR (qPCR) as described by Olvera and others [21].

### DNA extraction and qPCR to detect PCV2 nucleic acid in tissues and serum samples

In order to detect and to quantify the viral DNA by qPCR, DNA was firstly extracted from 200 μL of serum and 200 μL of 1:10 PBS homogenate of lymph node tissue, by using TRIzol LS (Invitrogen–San Diego, CA-USA) following the manufacturer’s instructions. The DNA obtained was suspended in 50 μL of DEPC-treated water. A qPCR was carried out using primers and probes according to Olvera and others [21] on a LightCycler 1.5 thermocycler (Roche Diagnostics–Basel, CH). The results were expressed as number of PCV2 genome copies per millilitre of serum or gram of tissue.

### Serology

#### Detection of PCV2 antibodies

For the detection of PCV2-specific antibodies, a commercially available indirect enzymatic immunoassay (INGENZIM CIRCO–INGENASA, Madrid, Spain) was used according to the manufacturer’s instructions. The titre of each sample was calculated as follows: titre = 53 (e^3.2×^) where **e** is the natural logarithm base and **x** is the S/P of the sample (S/P = sample O.D./positive control O.D.; O.D.: optical density).

#### Retrospective analysis of the courses of PCV2 antibodies after grouping the animals according to maternally derived antibodies at vaccination

The animals of the three replicates taken together were retrospectively grouped based on the value of the S/P ratio at PCV2 vaccination considering a high S/P value (≥ the median S/P ratio of all piglets at 4 weeks of age) and a low S/P value (< the median).

#### Detection of antibodies against other infections

Antibodies against *M. hyopneumoniae* were evaluated by a commercially available ELISA kit (Herd Check® *M. hyopneumoniae,* IDEXX Laboratories). The presence of antibodies against the gE glycoprotein of ADV was measured using a commercially available ELISA kit (HerdChek® PRV g1 (gE) test kit, IDEXX Laboratories) according to the manufacturer’s instructions. Another commercial ELISA kit (CHEKIT-APP-ApxIV ELISA test kit, IDEXX Laboratories) was used for the detection of antibodies against *A. pleuropneumoniae*. Antibodies to different SIV sub-types (H1N1, H1N2 and H3N2) were determined by a haemoagglutination inhibition (HI) assay, using a standard method [2]. The presence of antibodies to PRRSV were determined using a commercially available ELISA kit (HerdChek® Porcine Reproductive and Respiratory Syndrome Antibody Test Kit, IDEXX Laboratories, Westbrook, ME, USA) according to the manufacturer’s instructions.

### Quantification of PCV2-specific IFN-γ secreting cells

The frequencies of PCV2-specific IFN-γ secreting cells (SC) in the peripheral blood of pigs were determined according to Martelli and others [15] with minor modifications. Peripheral blood mononuclear cells (PBMCs) were isolated by Histopaque-1.077® gradient and plated at 4 × 10^5^ cells/well in complete RPMI-1640 (cRPMI-1640) supplemented with 10 % foetal bovine serum (FBS) into 96-well plates (MultiScreen®_HTS_-IP, Millipore) coated overnight at 4 °C with 5 μg/mL anti-pig IFN-γ mAb (P2G10, BD, Biosciences, Franklin lakes, NJ - USA) and blocked with cRPMI-1640 + 10 % FBS for 2 h at 37 °C. For the PCV2 antigen recall, an elaborate on the whole strain (I12/11) at 0.1 multiplicity of infection (MOI) was used as stimulus, in cRPMI-1640 + 10 % FBS, for 20 h at 37 °C, 5 % CO_2_. In all samples, >98 % of PBMCs were viable as confirmed by Trypan blue. Afterwards, plates were incubated for 1 h at 37 °C with 0.5 μg/mL anti-pig IFN-γ biotin-labelled mAb (P2C11, BD) and then with 1:750 AP-conjugated anti-biotin mAb in PBS + 0.5 % BSA. Plates were finally incubated for 7 min with a BCIP/NBT solution (BioRad, Hercules, CA - USA) and the reaction was stopped with distilled water. The frequencies of PCV2-specific IFN-γ SC were determined using an AID® ELISpot Reader (AID® ELISpot Software v.3.5 - AID). As a positive control, 4 × 10^5^ PBMCs/well were incubated with PHA (5 μg/mL); as a negative control, 4 × 10^5^ PBMCs were incubated without antigen (PCV2 mock stimulus: supernatant of non-PCV2-infected PK-15 cells). The background values (number of spots in negative control wells) were subtracted from the respective counts of the stimulated cells and the immune responses were expressed as number of IFN-γ SC per million PBMCs (IFN-γ SC/10^6^ PBMCs).

### Statistical analysis

All statistical analyses were carried out using the STATA/IC 12.1 for Windows (64-bit ×86-64; StataCorp LP, College Station, TX, USA). For all analyses, the individual pig was considered as the experimental unit.

Descriptive analysis was performed for all individual parameters from pigs stratified according to replicate and group. Outcome of continuous variables (ADWG, PCV2 ELISA S/P, IFN-γ SC and PCV2 viral loads) were tested for normality (Shapiro-Wild test) and further analyzed by either one way ANOVA (normally distributed data) or Kruskall–Wallis test (non-normally distributed data). Differences between groups were further evaluated by pairwise comparisons of marginal linear predictions. Moreover, piglets were re-grouped according to their level of MDAs into two groups: ‘Low level of MDA’ (S/P ratio < median S/P ratio of all piglets at 4 weeks of age) or ‘High level of MDA’ (S/P ratio ≥ median). A chi-square test was used to evaluate differences between groups in mortality, the number of animals with a PCV2 viral load higher than 10^6^ PCV2 copies/mL, and the number of animals needing an individual treatment. The correlation between ELISA S/P at the time of vaccination and four weeks later was tested employing linear regression analysis. The significance level (*α*) for all analyses was set at 0.05.

## Results

The results of this field trial are described for each of the three replicates, namely for pigs from nursery to the finishing phase and derived from the three consecutive litters (1^st^, 2^nd^ and 3^rd^ parity) of vaccinated sows.

### Morbidity and mortality

Morbidity in pigs of the three replicates is shown in Table 1. Among pigs of the first replicate, morbidity was significantly higher in those of groups C and D (p < 0.05). None of the recorded clinical signs that needed treatment was associated with or typical for PCVD. In the second replicate, a high proportion of treatments was needed in all groups and no statistical difference was observed. All the individual treatments were applied in the post-weaning period concomitantly with the occurrence of a severe outbreak of PRRSV infection and associated clinical signs. In the third replicate, morbidity in group B was significantly lower as compared to the other groups (p < 0.05). In particular, it is worth mentioning that the majority of the individual treatments in groups A, C and D were needed from 12 weeks of age until slaughter. Pigs belonging to group D had a very high percentage of diseased pigs (18 %) concomitantly with the high proportion of PCV2 viremic animals and high titres of viral burden (see the paragraph about PCV viremia) even if overt clinical signs compatible with PCV2-SD were not observed.

**Table 1:** Morbidity (parenteral injections) and mortality in the four groups under study for each replicate

	REPLICATE		GROUP			
			A	B	C	D
MORBIDITY*	1^st^		6.2^a^	5.4^a^	14.7^b^	18.9^b^
	2^nd^		22.7^a^	20.7^a^	21.3^a^	22.4^a^
	3^rd^		10.3^a^	0^b^	8.3^a^	18.2^a^
MORTALITY*	1^st^	from weaning to 12 weeks of age	3.1^a^	1.6^b^	3.1^a^	3.6^a^
		from 12 weeks to slaughter	3.9^a^	1.6^b^	4.7^a^	3.1^a^
	2^nd^	from weaning to 12 weeks of age	5.3^a^	4.0^a^	2.7^b^	4.3^a^
		from 12 weeks to slaughter	2.7^a^	2.7^a^	4.0^b^	5.4^b^
	3^rd^	from weaning to 12 weeks of age	5.7^a^	1.9^b^	2.0^b^	3.8^a^
		from 12 weeks to slaughter	14.0^a^	7.7^b^	18.0^a^	20.0^a^

Legend: * proportion of pigs; Different superscript letters indicate statistically significant differences (*p* < 0.05)

Group A: pigs vaccinated at 4 weeks; group B: pigs vaccinated at 6 weeks; group C: pigs vaccinated at 8 weeks; group D: non-vaccinated placebo/controls

Dead pigs, animals needing euthanasia, and runt non-marketable pigs (lost pigs) underwent pathological and virological investigations to be categorised as PCVD and non-PCVD affected. None of the investigated pigs fulfilled the diagnostic criteria for PCV2-SD. The mortality rate in the first replicate was significantly lower in group B (vaccinated at 6 weeks of age) (p < 0.05) in both of the considered periods of observation, namely from weaning to 12 weeks of age and from then to slaughter. In the second replicate, production cycle mortality was significantly lower in group C in the period from weaning to 12 weeks. In contrast, in the fattening period (from 12 weeks to slaughter), groups A and B had less mortality than other groups. None of the fatality cases fulfilled the diagnostic criteria of PCVD. In the last replicate, the mortality was significantly lower in groups B and C until 12 weeks and in group B only from 12 weeks of age to slaughter. The higher mortality in group D was associated with PCV2 viremia. Details are reported in the paragraph regarding PCV2 viremia.

### Average Daily Weight Gain (ADWG)

The ADWG is considered a useful parameter for measuring the effect of PCVD both in clinical and in subclinical cases. Table 2 shows the ADWG in vaccinated and placebo/control animals for the two intervals among the three different weighing time points. The ADWG of the four groups was not significantly different in any period of the three considered replicates.

**Table 2:** Average daily weight gain (ADWG) ± STD in the four groups under study for each replicate

	REPLICATE		GROUP			
			A	B	C	D
			(100)^b^	(100)^b^	(100)^b^	(100)^b^
ADWG^a^	1^st^	from weaning to 12 weeks of age	317 ± 78	303 ± 92	310 ± 69	325 ± 71
		from 12 weeks to 24 weeks of age	681 ± 129	649 ± 131	633 ± 98	683 ± 132
	2^nd^	from weaning to 12 weeks of age	244 ± 92	308 ± 72	255 ± 83	304 ± 75
		from 12 weeks to 24 weeks of age	613 ± 97	683 ± 115	623 ± 125	696 ± 104
	3^rd^	from weaning to 12 weeks of age	391 ± 72	410 ± 89	420 ± 91	363 ± 88
		from 12 weeks to 24 weeks of age	741 ± 110	740 ± 107	704 ± 124	730 ± 121

^a^average daily weight gain: grams/day; ^b^number of pigs at enrolment (first weighing)

Group A: pigs vaccinated at 4 weeks; group B: pigs vaccinated at 6 weeks; group C: pigs vaccinated at 8 weeks; group D: non-vaccinated placebo/controls

### PCV2 viremia

The course of PCV2 viremia was different in the three replicates. In the first replicate (litters from gilts, i.e. 1^st^ parity sows), PCV2 viremia was detected in some pigs of all groups (from 5 % to 25 %). The viral burden was always <10^6^ PCV2 genome copies/mL of serum. In the second replicate (litters from 2^nd^ parity sows), groups A, C and D had different proportions of viremic pigs, and the viral burden in vaccinated animals (groups A and C, but not B) and controls was confirmed to be always <10^6^ PCV2 genome copies/mL of serum. In the last replicate (litters from 3^rd^ parity sows), PCV2 viremia was detected in group D (placebo/control) from 16 weeks of age to the end of the experiment, and the proportion of viremic animals ranged from 47 % to 100 %. The high proportion of PCV2 viremia was associated with a viral burden >10^6^ PCV2 genome copies/mL of serum in at least one sampling per pig during the whole period of positivity.

### Serology

#### Serologic response to PCV2 vaccination and infection

**Fig. 1:**
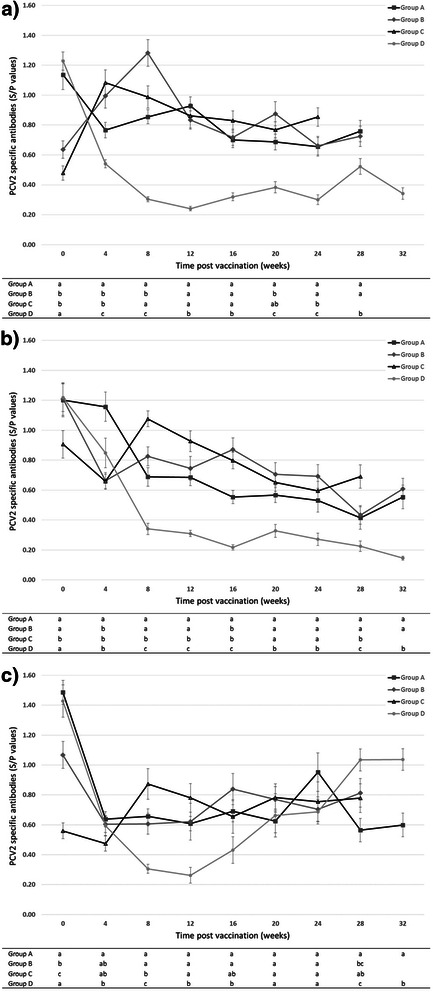
Course of the serologic response to PCV2 (Mean ± STD anti-PCV2 ELISA antibodies) in the four groups under study (PCV2-vaccinated and unvaccinated) for each consecutive replicate (**a**, **b**, **c**). Group A: pigs vaccinated at 4 weeks; group B: pigs vaccinated at 6 weeks; group C: pigs vaccinated at 8 weeks; group D: non-vaccinated placebo/controls

The course of serology for PCV2 in the three replicates is shown in Fig. 1 (a-c). In the first replicate, pigs of groups B and C had an increase of the S/P ratio after vaccination, whereas pigs in group A and non-vaccinated pigs showed a decline during the four weeks post-vaccination. In the second replicate, no increases of S/P values were recorded in any group after vaccination. From 12 weeks of age onwards in both replicates (1 and 2), all vaccinated groups showed significantly higher values of S/P compared to the controls (group D). The same course of ELISA antibodies in vaccinated pigs was observed in the third replicate. In fact, the values of the S/P ratio in vaccinated animals (all groups) did not increase after vaccination and kept a stable course over the threshold of positivity. At 16 to 18 weeks of age the non-vaccinated control pigs (group D) seroconverted shown by an increase of the average S/P ratio.

#### Retrospective analysis of the courses of PCV2 antibodies after grouping animals according to the levels of maternally derived antibodies at vaccination

**Fig. 2:**
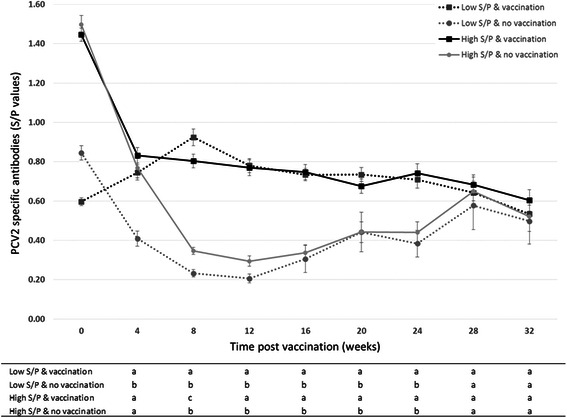
Course of the serologic response to PCV2 (Mean ± STD anti-PCV2 ELISA antibodies) according to the levels of maternal derived antibodies (MDA) at vaccination and PCV2 infection (viremia) occurred at 20 weeks of age. Overall, the animals of the 3 replicates were divided based on a low (≤0.9) or high (≥1.2) S/P value at 4 weeks of age when vaccinated animals were inoculated with the vaccine (“HIGH S/P vac” and “LOW S/P vac” groups) while unvaccinated animals were inoculated with the adjuvant alone (“HIGH S/P NV” and “LOW S/P NV” groups)

The values of the S/P ratio are also presented (Fig. 2). Animals with high S/P ratios due to MDA demonstrated an identical decline of PCV2 antibodies. In vaccinated animals, 4 weeks after vaccination, the decline stopped and remained in a stable state for the whole duration of the study. Conversely, in non-vaccinated animals having high S/P values, the decline was longer reaching values below the threshold of seropositivity (<0.4) at 12 weeks. The animals with low S/P values at vaccination showed no decline of antibodies and a stable course that was not significantly different compared to the levels in vaccinated pigs with high S/P values. In the low S/P value non-vaccinated group, the levels of antibodies declined promptly, reaching a negative value in 4 weeks. Similarly to the non-vaccinated group with high S/P value, the viremic pigs of this group seroconverted later in their life.

The antibody course in non-vaccinated groups declined and maintained values below the threshold of seropositivity for the whole duration of the study. In contrast, vaccination in animals with high S/P values did not induce seroconversion but only a decline followed by a stable state. In animals with low S/P values, the course of antibodies was characterized by a marked seroconversion and the S/P values shown for the whole duration of the trial were not different as compared to those of animals with high S/P values at vaccination.

A correlation between S/P values at the time of vaccination and four weeks later could be confirmed for pigs in group A and group D (Table 3). In contrast to this, no correlation were observed when pigs were vaccinated with 6 or 8 weeks of age.

**Table 3:** Correlation of PCV2-specific antibodies in piglets at the time of vaccination and 4 weeks later in the four groups under study for each replicate

	Regression coefficient	Lower CI	Upper CI	p-value
Group A	0.521	0.218	0.825	<0.001
Group B	−0.088	−0.474	0.299	0.651
Group C	0.038	−0.215	0.290	0.766
Group D	0.777	0.500	1.055	<0.001

Group A: pigs vaccinated at 4 weeks; group B: pigs vaccinated at 6 weeks; group C: pigs vaccinated at 8 weeks; group D: non-vaccinated placebo/controls

#### Serologic response to other infections

Serologic investigations performed to monitor the most frequently occurring infections in the herds (PRRSV and *M. hyopneumoniae*) found that the prevalence of PRRSV infection was 100 % at 12-15 weeks of age in all groups and replicates. Concomitantly, *M. hyopneumoniae* seroconversion was also observed. For the latter antigen, seroprevalence continued to increase in the subsequent period in all replicates (data not shown).

Low titres of antibodies (at HI) to SIV were detected in some samples and were inconclusive. Antibodies to ADV were not detected in any sample at any time point.

### Cell-mediated immune response to PCV2 vaccination and infection

**Fig. 3:**
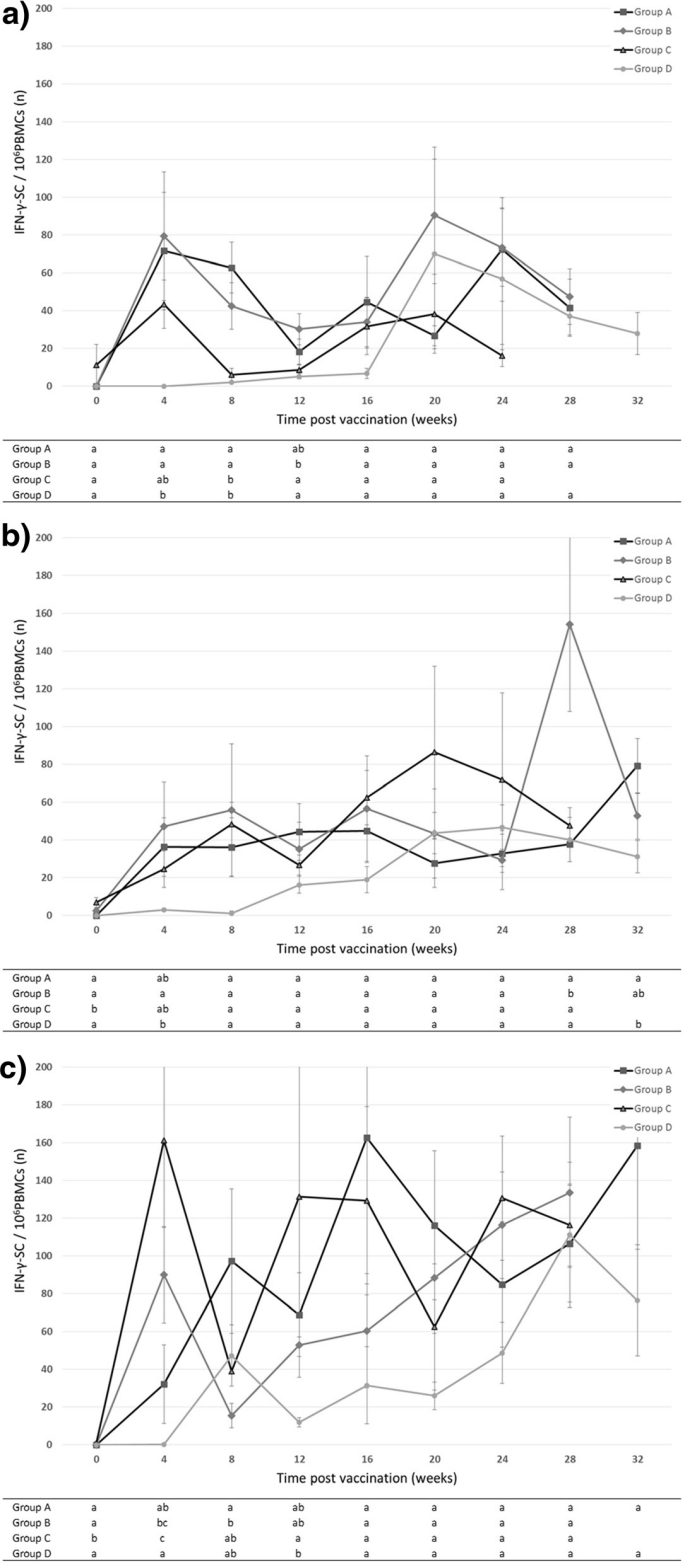
Course of PCV2-specific IFN-γ SC number (Mean ± STD) in the four groups under study (PCV2-vaccinated and unvaccinated) for each consecutive replicate (**a**, **b**, **c**). Group A: pigs vaccinated at 4 weeks; group B: pigs vaccinated at 6 weeks; group C: pigs vaccinated at 8 weeks; group D: non-vaccinated placebo/controls

In the first and second replicates, in all vaccinated groups, an increase of the PCV2-specific IFN-γ SC levels was observed after vaccination regardless of the vaccination timing (4, 6 or 8 weeks of age). For the whole duration of the study, the frequencies of IFN-γ SC remained stable with some non-significant variations. Control animals (group D) concomitantly showed an increase with PCV2 infection in some animals. Notably, in the second replicate, the values of IFN-γ SC after vaccination were lower as compared to those recorded in the first replicate (Fig. 3a-c).

In the third replicate, the increase of IFN-γ SC post-vaccination was numerically higher in comparison with the previous replicates. Moreover, the IFN-γ SC maintained high levels during the whole period of infection concomitantly with the onset and increase of the response in the control group (group D) between 20-22 and 34 weeks of age.

## Discussion and conclusions

The present study is aimed at assessing the efficacy of and the immunological response to a commercial PCV2a sub-type based subunit vaccine when administered at different ages (4, 6 and 8 weeks of age) in pigs with theoretically higher and more homogeneous levels of maternally derived immunity obtained by repeated vaccinations in the population of the matching mothers (gilts and sows). Thus, this purpose could allow defining and evaluating a delayed vaccination scheme of piglets (vaccination at 6 or 8 weeks of age) in respect to the conventional timing (3 weeks of age or earlier) in order to optimize the vaccination scheme. To evaluate the effects of vaccination, clinical, pathological and virological outcomes as well as some aspects of the humoral and cell-mediated immune response towards PCV2 were studied. Under the conditions of this study, the onset of the clinical signs related to PCV2-SD did not occur in the first two replicates as demonstrated by the relatively low PCV2 natural challenge in the blood of vaccinated and particularly of non-vaccinated and more susceptible pigs. In the third replicate, PCV2 infection and morbidity were detected from 16 weeks of age to the end of the observation period in controls in conjunction with a more intense PCV2 natural challenge.

The present data clearly indicates that vaccination against PCV2 at different ages induces a statistically significant reduction of the proportion of viremic animals and also of the viral load in the blood compared to placebo/controls (p < 0.05).

Vaccination with a single dose of the test vaccine administered intramuscularly at 3 weeks of age consistently reduced the clinical signs attributed to PCVD as well as mortality and PCV2 viral load and viremia [7, 15, 16]. More recently, the effect of sow and piglet vaccination under the same field conditions was proven [9].

Overall in the three replicates, it was confirmed that mortality was numerically reduced in vaccinated animals when PCV2 infection occurred and, under the conditions of this study, statistically significant differences were observed in animals vaccinated at 6 weeks of age. To measure morbidity, the number of individual treatments in unvaccinated and vaccinated animals was recorded throughout the study. After intense PCV2 natural challenge, morbidity was significantly higher in the placebo/control group compared to all vaccinated animals.

Average daily weight gain was also evaluated as an efficacy parameter for the vaccination schedule under investigation. Both before the onset of PCV2 viremia and associated disease (from 4 to 12 weeks of age), and from 12 to 24 weeks (the period when PCV2 viremia occurred in the third replicate), the ADWG was not positively affected in vaccinated animals. This result is not in accordance with previous studies [4, 12, 14, 15, 27] supporting a positive effect of vaccination on this parameter. The inability to detect any influence of PCV2 viremia on the productive performance of vaccinated animals compared to controls can be attributed to the early measurement of body weight (24 weeks of age) in respect to the occurrence of viremia detected until the end of the study. Thus control animals experienced the effects of PCV2 viremia after the last weighing. These results highlight the fact that PCV2 natural challenge can occur late in the fattening period and, particularly, that viremia can last very long until the slaughter time.

The improved clinical parameters (morbidity and mortality) are associated with a significant reduction in the proportion of infected pigs and in the viral load in the blood. Particularly, in the third replicate, when PCV2 natural challenge overtly occurred (from 16 weeks of age onward), a high number of placebo/control pigs had high viral loads in serum (>10^6^ DNA copies/mL), whereas in vaccinated pigs the duration of viremia and the viral load were markedly lower (A and C) or neglectable (group B). It is remarkable that pigs vaccinated at 6 weeks of age (group B) never became PCR-positive for PCV2 in all replicates. It is also notable that, in an almost one year and half time period of observation in the field conditions of this study, PCV2 infection was undetectable for a certain time period (two replicates) and suddenly re-appeared without any evident and expected reason.

All tested animals were PCV2 negative by PCR at the time of vaccination so that the antibody titres detected were most likely of maternal origin also in pigs vaccinated at 8 weeks of age. Overall, the S/P ratio in piglets at 4 weeks of age increased in the three subsequent replicates as a consequence of the effect of repeated vaccination of the corresponding dams at mating. Thus, in the 1^st^ replicate, even if the S/P ratios were the lowest within the three considered replicate, no seroconversion was measured overall in piglets vaccinated at 4 weeks of age as a consequence of some interference of the MDA passively transferred by colostrum on seroconvertion by vaccination. This result is in accordance with a previous study [15]. Conversely, seroconversion could be measured in animals vaccinated at 6 and 8 weeks of age (group B and C, respectively) as a direct consequence of the decline of MDA occurring within 2-4 weeks. In the 2^nd^ and 3^rd^ replicates this reduction of MDA was delayed because of the higher level of the S/P ratio at 4 weeks of age. However, upon vaccination, even when seroconversion did not occur, the S/P ratios remained above the threshold of positivity (S/P >0.4) throughout the study. On the other hand, the placebo/controls had a continuous decline of MDA reaching negative values (S/P <0.4) at 10 weeks of age. The retrospective analysis of the course of PCV2 antibodies after grouping animals according to maternally derived antibody levels measured as S/P ratios at vaccination supports that animals with high values of S/P ratio do not seroconvert whereas vaccinated animals with low S/P values do. Interestingly, the course of the S/P ratios for the whole duration of the study maintained at stable levels with no differences in the absolute values comparing the two groups (high S/P vs. low S/P). Moreover, non-vaccinated animals, both with high and low MDA, experience a decline reaching negative values during the second month of life. Non-vaccinated animals grouped according to the occurrence of PCV2 natural challenge seroconvert soon after viremia. Vaccinated animals either seroconverting because of low levels of MDA or not due to high MDA, maintain stable and efficient, protective values of antibodies for the whole production cycle even when PCV2 natural exposure occurs relatively late in their life (from 16 weeks onward). Thus, these results make clear that the degree of protection is not higher in animals with low S/P values at vaccination compared to those with high values. It has been shown that in Group A and D antibody concentration at the time of vaccination significantly correlates with the antibody concentration four weeks later, which was always lower than the value before. This underlines the hypothesis that high concentration of MDA prevent seroconversion in vaccinated pigs (Group A), whereas low to medium concentration (Group B and C, being two and four weeks, respectively, older at the time of vaccination) does not interfere with the humoral immune response. The latter assumption of no correlation between these levels of MDA and the individual response to vaccination is confirmed by the absence of any significant correlation of S/P values in Group B and C during four weeks following vaccination.

Some studies investigated the influence of age on the efficacy of one-dose PCV2 vaccination in piglets with high levels of maternally derived antibodies. A recent paper studied the efficacy of vaccination at one and three weeks of age under field conditions [11]. Similarly to this study, we detected mean values of MDA in piglets that increased in parallel with the parity of the sows [17].

A previous study [15] demonstrated that PCV2 vaccine efficacy is also sustained by the development of the cellular immune response and this is confirmed by the increased levels of IFN-γ SC in pigs soon after vaccination, supporting the capacity of the PCV2 subunit vaccine to induce cellular immunity which response is not negatively influenced by the presence of high MDA. The age-dependent induction of the cellular response and the influence of the MDA in vaccinated animals are important features to evaluate since different outcomes (extent and duration of the recalled immunity) can be observed in different species and in relation to the challenge conditions [1, 3, 24, 25]. The data of the present paper confirm that the cell-mediated immunity to PCV2 measured as IFN-γ SC was induced within 4 weeks post-vaccination in all replicates and all vaccinated groups. The levels of IFN-γ SC are maintained stable throughout the study (34 weeks of age) and are at the highest levels during the period of late infection that occurred in the third replicate.

In conclusion, the present study demonstrates the beneficial effect of vaccination with a single dose of a PCV2 Cap-based vaccine against PCVD under field conditions even in the presence of high levels of MDA at the time of vaccination, particularly when the experimental animals (all groups) were exposed to a “robust” PCV2 natural infection.

The effects of vaccination at different ages are strictly associated with virological and clinical protection as a result of the immunogenicity of the tested vaccine measured as activation of both the humoral and cellular immune response. This trial demonstrates that even in a condition of high levels of MDA, piglets vaccination at 4, 6 or 8 weeks of age confer a protective immune response characterized by cellular immunity and a stable and long-lasting (until 34 weeks of age) antibody response. However, in the conditions of this study, the combination of vaccination in sows at mating and in piglets at 6 weeks of age was more effective for controlling PCV2 natural infection, than other treatment schemas, thus sustaining that some interference of MDA with the induction of an efficient immune response could be considered. These evidences are in agreement with the conclusion of a research based on experimentally PCV2 infected pigs with or without MDA [20]. In conclusion, optimal vaccination strategy needs to balance the levels of passive immunity, the management practices and timing of infection.

### Ethics

Ethics is mentioned in the text.
